# Development of hepatopancreatic arterial-dominant phase (HPAP) images using partial downslope injection technique and application to evaluation of pancreatic ductal adenocarcinoma

**DOI:** 10.1016/j.ejro.2026.100793

**Published:** 2026-07-06

**Authors:** Takayuki Yokota, Tomoaki Ichikawa, Hiroyuki Yasui, Koji Muroga, Yoshito Tsushima

**Affiliations:** a Department of Diagnostic Radiology and Nuclear Medicine, Gunma University Graduate School of Medicine, 3-39-22 Showa-machi, Maebashi, Gunma 371-8511, Japan; b Department of Diagnostic Radiology, Nagano Red Cross Hospital, Wakasato 5-22-1, Nagano, Nagao 380-8582, Japan

**Keywords:** CT, Pancreas, Pancreatic ductal adenocarcinoma, Downslope, Contrast enhancement

## Abstract

**Background:**

There is frequently ambiguity regarding the optimal utilization of the liver protocol or the pancreas protocol, and the selection between HAP and PPP is often the subject of controversy. The purpose of this study is to evaluate the validity and efficacy of newly developed hepatopancreatic arterial-dominant phase (HPAP) imaging, which synchronizes HAP and PPP using the partial downslope injection technique (pDiT), compared with conventional HAP and PPP in detecting pancreatic ductal adenocarcinoma.

**Materials and methods:**

This retrospective study included 130 examinations with pancreatic ductal adenocarcinoma who underwent pancreatic CT using either pDiT or a standard injection technique between November 2023 and June 2025. The injection duration was 30-second. The pDiT used 360 mgI/kg at 80 kVp, consisting of a 10-second constant injection followed by a 20-second downslope injection with a slope index (end injection rate / initial injection rate) of 0.3. The standard injection technique used 600 mgI/kg at 120 kVp with a constant rate. Pancreas-to-lesion contrast (PLC), contrast-to-noise ratio (CNR), lesion conspicuity, and sensitivity were compared among HAP, PPP, and HPAP.

**Results:**

Mean PLC and CNR were significantly higher with HPAP than with HAP (*p* = .03 and *p* = .04, respectively) and comparable to PPP (*p* = .79 and *p* = .94, respectively). Lesion conspicuity showed no significant difference among the three (*p* = .30). Lesion sensitivity with HPAP was significantly higher than with HAP (*p* = .03) and not significantly different between HPAP and PPP (*p* = .56).

**Conclusion:**

HPAP generated using the pDiT provides optimal arterial-phase imaging for pancreatic CT.

## 1 Introduction

Multiphasic contrast-enhanced CT (CE-CT) is an essential imaging tool in the management of pancreatic cancers [1], [2]. Particularly, it has been well recognized that pancreatic parenchymal phase (PPP) images, by maximizing contrast enhancement (CE) of the pancreatic parenchyma, play a major role in detecting hypovascular, conventional pancreatic cancer [3], [4], [5]. On the other hand, multiphasic CE-CT plays an important role in the evaluation of the liver as well. In the assessment of liver tumors, the evaluation of hypervascular tumors, particularly in the hepatic arterial-dominant phase (HAP) images, is of particular importance [6], [7]. The optimal timing for HAP images is generally considered to be approximately 35–40 s after the start of contrast material (CM) injection [8]. If the scan is performed later than the optimal timing, the contrast between the tumor and the liver parenchyma decreases [8], [9]. Previous studies have reported that the optimal scan timing for PPP images is approximately 5–10 s later than the optimal scan timing for HAP images [10]. In the context of actual imaging, there is often ambiguity regarding the optimal utilization of the liver protocol or the pancreas protocol, and the selection between HAP and PPP is frequently the subject of controversy.

We have recently developed downslope injection technique (DiT), with continuously decelerated injection rate of CM. Compared to standard injection techniques with a constant injection rate of CM, DiT can significantly increase the peak CE value of the aorta, resulting in improving CE of hypervascular liver lesions on the HAP images [11]. Consequently, it has been reported that DiT significantly improved the detectability of hypervascular liver lesions [11]. They have also emphasized another advantageous effect of DiT that the peak CE time of the aorta appears earlier, advancing CE time of the hypervascular liver lesions [11]. Based on this effect, DiT should also advance the peak CE time of the pancreatic parenchyma, which is a representative hypervascular tissue, shifting the PPP optimum earlier. As a result, images obtained with DiT at the same timing for the standard HAP images would yield sufficient CE of the pancreas similar to that on the standard PPP images, while maintaining the suitable image contrast of HAP images in the liver—a phase we term hepatopancreatic arterial-dominant phase (HPAP). The evaluation of pancreatic ductal adenocarcinoma on HPAP images is presumed to be comparable to that on PPP; therefore, when considering pancreatic ductal adenocarcinoma alone, HPAP does not offer any clear advantage. On the other hand, we believe that the ability to simultaneously evaluate hypervascular lesions is useful for the screening of pancreatic tumors of undetermined nature. In other words, in the present study, we believe it is important to establish a phase that allows for the simultaneous evaluation of hypervascular and hypovascular lesions by examining whether arterial-phase images acquired using DiT—which has been reported in a previous study to improve the diagnostic performance of hypervascular (hepatic) tumors—are equivalent to PPP in terms of diagnostic performance for pancreatic ductal adenocarcinoma, a representative hypovascular pancreatic tumor. The purpose of this study is to assess the validity and the efficacy of the HPAP images obtained with DiT compared to the conventional HAP and the PPP images obtained with the standard injection technique in the detection of conventional pancreatic cancers.

## 2 Materials and methods

### 2.1 Study sample

This study was approved by our institutional review board. Because this study was a retrospective study and the images were completely anonymized, the requirement for informed consent was waived.

**Fig. 1:**
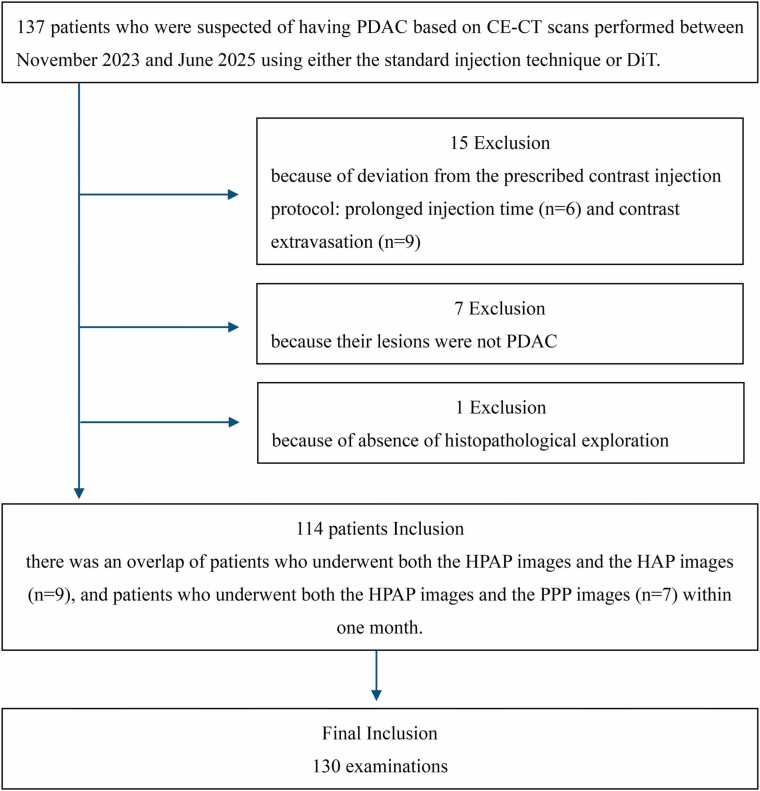
Inclusion and exclusion criteria flowchart. PDAC= pancreatic ductal adenocarcinoma, DiT= downslope injection technique, HPAP= hepatopancreatic arterial-dominant phase, HAP= hepatic arterial-dominant phase, PPP= pancreatic parenchymal phase.

We identified patients at our institution who were suspected of having pancreatic ductal adenocarcinoma (PDAC) based on CE-CT scans performed between November 2023 and June 2025 using either the standard injection technique or DiT. Exclusion criteria were: (1) deviation from the prescribed injection protocol of CM (e.g., prolonged injection time due to increased injection pressure or extravasation of CM); (2) lack of histopathologic confirmation of PDAC. The patient selection process is shown in Fig. 1.

### 2.2 Image acquisition

All CE-CT examinations were performed on one of the following commercially-available CT scanners: Revolution HD (GE Healthcare, WI, USA), Aquilion ONE (Canon Medical Systems, Otawara, Japan) or SOMATOM Definition Flash (Siemens Healthcare, Erlangen, Germany). CT images were reconstructed with a slice thickness of 3 mm and an interval of 3 mm. CT images acquired using Revolution HD were obtained with automatic tube current modulation (noise index of 12), pitch of 0.985 and collimation of 0.625 mm, and were reconstructed using adaptive statistical iterative reconstruction-V (ASiR-V, 20%) with a standard soft-tissue kernel. Images acquired using SOMATOM Definition Flash were obtained with automatic tube current modulation (Quality reference mAs of 350), pitch of 0.700 and collimation of 0.6 mm, and were reconstructed using sinogram-affirmed iterative reconstruction (SAFIRE, strength 2) with an I31f kernel. Images acquired using Aquilion ONE were obtained with automatic tube current modulation (standard deviation of 13), pitch of 0.813 and collimation of 0.5 mm, and were reconstructed using adaptive iterative dose reduction 3D (AIDR 3D, eMILD) with an FC13 kernel. HPAP images were acquired using Revolution HD or Aquilion ONE. All patients received a body-weight-tailored iodine dose (Optiray, 240 or 320 mgI/mL, Guerbet-Japan, Tokyo, Japan; Omnipaque, 300 or 350 mgI/mL, GE HealthCare Pharma, Tokyo, Japan; Iomeron, 300 or 350 mgI/mL, Bracco-Japan, Tokyo, Japan; Iopamiron, 370 mgI/mL, Bayer Yakuhin, Osaka, Japan or Iopamidol, 370mgI/mL, Hikari Pharmaceutical, Tokyo, Japan) via a commercially-available power injector (Dual Shot GX: Nemoto Kyorindo, Tokyo, Japan).

The iodine dose and tube voltage were set at 360 mgI/kg and 80 kVp for the DiT, and at 600 mgI/kg and 120 kVp for the standard injection technique. With DiT, the CM injection rate at the start of administration is higher than with the standard injection technique; therefore, since it was difficult to use a CM of 600 mgI/kg, the iodine dose was reduced to 360 mgI/kg, and imaging was performed at 80 kVp to ensure equivalent contrast based on previous reports [12], [13]. The injection duration was similarly fixed for the 30 s with both injection techniques. The standard injection technique employed a constant injection rate during the injection duration automatically determined from a combination of the total iodine dose and the injection duration in each patient.

**Fig. 2:**
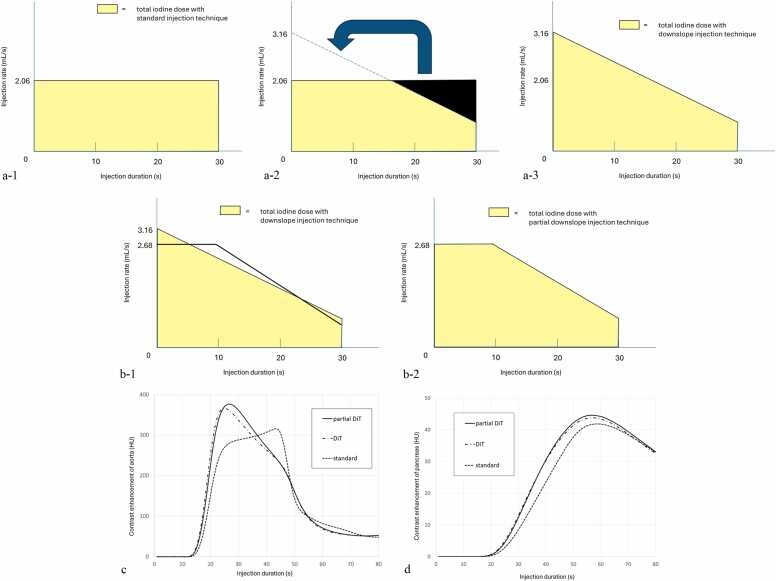
Principle of Partial Downslope Injection Technique. The figures are created with a computer-simulation model software based on a patient model with following conditions; body weight = 60 kg, total iodine dose = 360 mgI/kg, concentration of contrast material (CM) = 350mgI/kg, injection duration = 30 s, slope index (SI) = 0.3. The standard injection technique typically employ a constant injection rate (Figs. 2a-1). While the original downslope injection technique (DiT) increases the initial injection rate and continuously decelerates it over the injection time without changes of total iodine dose and the injection duration compared to those with the standard injection technique (Figs. 2a-2). The degree of the deceleration of the injection rate (slope on the figures) is determined by establishment of SI defined by a following equation; injection rate at the end of initiation of injection of CM / injection rate at the start of initiation of injection of CM. Major problem of the original DiT is that an initial injection rate may sometimes become fast exceeding a clinically-permissible injection rate: It becomes approximately 1.5 times of that with the standard injection technique (1.53 times (3.16/ 2.06 mL/ s) on Figs. 2a-3). The partial DiT is developed for the purpose of reducing an initial injection rate with the original DiT and consists of a combination of a constant injection rate for the first 10 s and the subsequent downslope injection (SI= 0.3) for 20 s (Figs. 2b-1). The partial DiT can further suppress an initial injection rate by approximately 1.3 times of that with the standard injection technique (1.30 times (2.68/ 2.06 mL/ s) on Figs. 2b-2). On time-enhancement curves (TEC) of the aorta with the standard injection technique (shown in dotted line) and the original (shown in dashed line) and the partial (shown in solid line) DiT (Fig. 2c), profiles of TEC similarly changes and the peak contrast enhancement (CE) of the aorta shifts earlier and higher compared to that with the standard injection technique (shown in dotted line). Moreover, the peak CE value of the aorta with the partial DiT becomes even higher than that with the original DiT. Consequently, TEC of the pancreas (Fig. 2d), which is a representative hypervascular tissue, shifts earlier as well as that of the aorta with the original/ partial DiT, in which a given CE value of the pancreas appears approximately 5 s earlier than that with the standard injection technique.

**Fig. 3:**
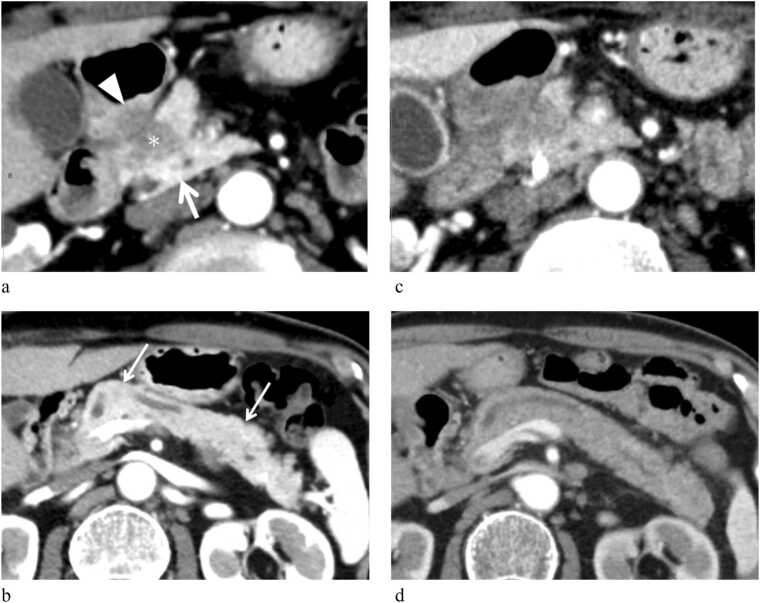
This is a case of pancreatic cancer of the head of the pancreas with duodenal invasion in a man in his 60 s. (a-d): Transverse contrast-enhanced hepatopancreatic arterial-dominant phase (HPAP) images with the partial downslope injection technique (a, b) and hepatic arterial-dominant phase (HAP) images with the standard injection technique (c, d) obtained at the same scan timing of 40 s after the initiation of injection of contrast material. All images are displayed with 300/70 of window width/ level. (a, c) The HPAP images show greater contrast enhancement (CE) of the head of the pancreas (bold arrow) and pancreas-to-lesion (asterisk) contrast (182 HU and 93 HU, respectively) than the HAP image (104 HU and 36 HU, respectively). The lesion conspicuity was also judged better for the HPAP image (visual score; 5) than for the HAP image (visual score; 4). Moreover, the lesion extension to the duodenal wall (arrowhead) can be more clearly recognized on the HPAP image because CE of the duodenal wall is greater on the HPAP image than on the HAP image. (b, d) CE of the body and the tail of the pancreas (thin arrows) may also preserve as well as that of the head of the pancreas on the HPAP image, while it is seriously diminished on the HAP image because of the existence of duct-obstructive pancreatitis.

**Fig. 4:**
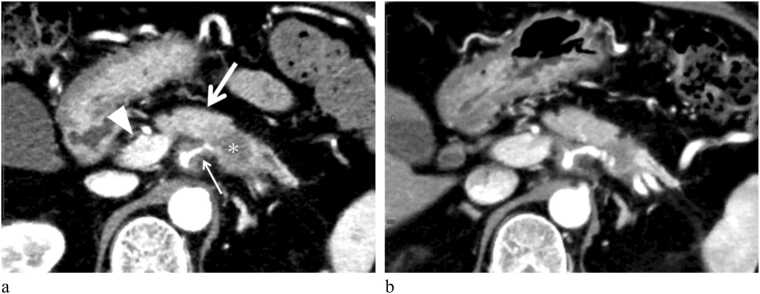
This is a case of pancreatic cancer of the tail of the pancreas with celiac arterial invasion in a man in his 50 s. (a, b): Transverse contrast-enhanced hepatopancreatic arterial-dominant phase (HPAP) image with the partial downslope injection technique (a) and pancreatic parenchymal phase (PPP) image with the standard injection technique (b) obtained at scan timing of 40 s (a) and 45 s (b) after the initiation of injection of contrast material. All images are displayed with 300/60 of window width/ level. (a, b) The lesion conspicuity (asterisk) was equally judged better for both HPAP and PPP image (visual score; 5) although both contrast enhancement (CE) of the pancreas (bold arrow) and pancreas-to-lesion (asterisk) contrast tended to be greater on the HPAP image (136 HU and 72 HU, respectively) than on the PPP image (128 HU and 59 HU, respectively). The celiac arterial encasement (thin arrow) and CE of the portal vein (arrowhead) can be also demonstrated clearly and equally on both the HPAP and the PPP images.

The DiT employed in the present study was partial DiT (pDiT), which was improved from the above-mentioned original DiT [11]. The original DiT with a slope index (SI = final injection rate/ initial injection rate) of 0.3 increases the initial injection rate and continuously decelerates it over the injection time without changing total iodine dose with the standard injection technique (Fig. 2a), resulting in realizing earlier and higher peak CE of the aorta than the standard injection technique does [11]. The main disadvantage of the original DiT is that its initial injection rate becomes approximately 1.5 times as fast as that with the standard injection technique, resulting in excessively high rates in some cases. The pDiT consists of a combination of the standard injection with a constant injection rate for the first 10 s and the subsequent downslope injection (SI= 0.3) for 20 s, without changing both the total iodine dose and the injection duration (Fig. 2b). Due to such improvement, the pDiT can further suppress an initial injection rate by approximately 1.3 times of that with the standard injection technique and shows higher peak CE value of the aorta even compared to that with the original DiT in computer-simulation models [14] (Fig. 2c). Consequently, the pDiT has similar effects on CE of the pancreas to the original DiT, and a given CE value of the pancreas with the pDiT appears approximately 5 s earlier than that with the standard injection technique in computer-simulation models [14] (Fig. 2d). As a result, high contrast enhancement of the pancreatic parenchyma can be achieved at the same timing as HAP, which is useful for evaluating hypervascular liver tumors. While the optimal timing for the arterial phase in the liver and pancreas differed slightly with the standard injection technique, the application of pDiT enabled the evaluation of both the liver and pancreas in a single imaging phase; The term employed for such images is the hepatopancreatic arterial-dominant phase (HPAP) images (Fig. 3, Fig. 4).

The HPAP images with the pDiT and the HAP images with the standard injection technique were similarly acquired 10 s after the end of CM injection, while the PPP images with the standard injection technique were acquired 15 s after the end of CM injection, respectively.

At our institution, when ordering a CE-CT examination, either the liver protocol or the pancreas protocol is selected. Based on this selection, we used the standard injection technique to acquire HAP images under the liver protocol. In the context of the pancreas protocol, pDiT was utilized for the acquisition of HPAP images; However, in instances where pDiT was not available on the injector (although unrelated to the present study, the utilization of pDiT was suspended from May to June 2025, with the objective of effecting an adjustment to the imaging protocol), the standard injection technique was employed for the acquisition of PPP images for cases in which the pancreatic protocol was selected during this period.

### 2.3 Quantitative evaluation

One radiologist in our institute (T.Y. with 7 years of abdominal CT experience) performed measurements of CT numbers of aorta, portal vein, pancreatic parenchyma, and the lesions, and standard deviation (SD) of paraspinal muscle.

The regions of interest (ROIs) were placed on the structure or organ, based on an agreement to make it as large as possible. ROIs in the pancreatic parenchyma and lesions were placed to carefully exclude peripancreatic adipose tissue, vasculature, intralesional necrosis (if present) and image artifacts. Contrast enhancement (CE), pancreas-to-lesion contrast (PLC: CT number of pancreatic parenchyma – CT number of lesion) and contrast-to-noise ratio (CNR: PLC / image noise (SD of paraspinal muscle)) were derived from these measurements.

### 2.4 Qualitative evaluation

Image interpretation for all images was independently performed by three radiologists (T.I., H.Y. and T.Y. with 33, 7 and 7 years of abdominal CT experience, respectively). They were blinded to image type, clinical course, and histopathologic evaluation. They rated lesion conspicuity on a 5-point Likert scale (visual score). The evaluation criteria for the Likert scale in the present study were as follows: 5 = the lesion was clearly visible, and we were confident that a hypovascular pancreatic lesion was present, 4 = there was likely a hypovascular pancreatic lesion, but this was not certain, 3 = it was difficult to determine whether a hypovascular pancreatic lesion was present, 2 = there was likely no hypovascular pancreatic lesion, 1 = we were confident that a hypovascular pancreatic lesion was absent. Lesions classified as grades 4 or 5 were considered positive for analyzing lesion detectability.

### 2.5 Statistical analysis

Continuous variables are expressed as mean ± standard deviation. Because some subjects were included in more than one group, resulting in non-independent observations, a linear mixed-effects model was applied to compare CE, PLC, and CNR among the three different injection technique groups. The group was included as a fixed effect, and subject identity was modeled as a random effect to account for within-subject correlation.

Categorical outcomes were analyzed using generalized linear mixed-effects models to appropriately account for within-subject correlation arising from partially overlapping observations across groups. For lesion conspicuity assessed on a 5-point Likert scale, cumulative logit mixed-effects models were used. For lesion sensitivity, mixed-effects logistic regression models with a logit link function were fitted. In each model, group was included as a fixed effect, and subject identity was modeled as a random effect to account for repeated or correlated measurements within the same subject. The significance of the overall group effect was evaluated using a likelihood ratio test by comparing the full model with a reduced model excluding the group term for categorical outcomes. When a significant overall group effect was detected, post hoc pairwise comparisons were conducted with adjustment for multiple comparisons using the Holm method. A P value less than 0.05 was considered statistically significant. Statistical analyses were performed using EZR [15].

The degree of interobserver agreement was calculated using the Fleiss kappa statistic. In general, a kappa statistic of 0.81–1.00 indicates near perfect agreement, 0.61–0.80 substantial agreement, 0.41–0.60 moderate agreement, 0.21–0.40 fair agreement, 0.01–0.20 slight agreement, and agreement below 0 is considered poor [16].

## 3 Results

### 3.1 Characteristics of the study sample

During the study period, 137 patients with suspected PDAC were identified. 15 patients were excluded because of deviation from the prescribed contrast injection protocol (6 patients with prolonged injection time, and 9 patients with contrast extravasation). 7 patients were excluded because their lesions were not PDAC and 1 patient was also excluded due to the absence of histopathological exploration. Consequently, 114 patients were included in the analysis. Within the present study, there was an overlap of 9 patients who underwent both the HPAP images and the HAP images, and 7 patients who underwent both the HPAP images and the PPP images within one month. Finally, 130 CT examinations having a pancreatic cancer were included in the present study: 52 with the HPAP images (24 men, 28 women; mean age 72.3 years; mean body weight 54.0 kg; mean lesion size 27.6 mm), 47 with the HAP images (22 men, 25 women; mean age 70.9 years; mean body weight 54.3 kg; mean lesion size 25.9 mm), and 31 with the PPP images (12 men, 19 women; mean age 73.3 years; mean body weight 51.1 kg; mean lesion size 24.6 mm). All 31 cases for which PPP images were obtained were acquired during the period when the utilization of pDiT had been suspended. Therefore, the pancreatic protocol includes both the HPAP and the PPP images. All of 114 lesions had histopathological confirmation of pancreatic ductal adenocarcinoma in 43 cases through surgical specimens and in 71 cases through fine needle aspiration (FNA) biopsy. No significant differences in demographics were noted among the three techniques. (Table 1).

**Table 1:** Participant Demographics and Characteristics.

Participant Demographics/Characteristics				
	HPAP	HAP	PPP	P value
Male/Female	24/28	22/25	12/19	.75
Age (y)	72.3 ± 9.9	70.9 ± 11.6	73.3 ± 8.4	.59
Body weight (kg)	54.0 ± 7.4	54.3 ± 8.5	51.1 ± 8.8	.18
Lesion diameter (mm)	27.6 ± 12.0	25.9 ± 10.8	24.6 ± 11.6	.50

Note. -- Data presented as mean ± standard deviation. HPAP= hepatopancreatic arterial-dominant phase, HAP= hepatic arterial-dominant phase, PPP= pancreatic parenchymal phase.

**Table 2:** Mean Contrast Enhancement Values of Each Organ and Lesion, Pancreas-to-Lesion Contrast, and Contrast-to-Noise Ratio on HPAP Images with Partial Downslope Injection Technique and HAP and PPP Images with Standard Injection Technique.

Quantitative Analyses						
				p value		
	HPAP	HAP	PPP	Overall	HPAP vs HAP	HPAP vs PPP
Image noise	9.8 ± 1.6	9.6 ± 1.0	10.3 ± 1.8	.19	N/A	N/A
CE						
Aorta (HU)	273.4 ± 58.7	285.3 ± 77.4	277.2 ± 83.3	.92	N/A	N/A
Portal vein (HU)	154.8 ± 47.1	132.1 ± 40.0	136.2 ± 40.0	< .01	< .01	.02
Pancreatic Parenchyma (HU)	95.2 ± 20.3	79.8 ± 17.5	89.1 ± 15.9	< .01	< .01	.09
Lesion (HU)	33.9 ± 16.1	29.1 ± 12.7	28.7 ± 15.4	.06	N/A	N/A
PLC (HU)	66.0 ± 25.4	53.8 ± 14.7	65.1 ± 20.6	.02	.03	.79
CNR	6.8 ± 2.6	5.6 ± 1.4	6.5 ± 2.3	.04	.04	.94

Note. --Data presented as mean ± standard deviation. HPAP= hepatopancreatic arterial-dominant phase, HAP= hepatic arterial-dominant phase, PPP= pancreatic parenchymal phase, CE= contrast enhancement, PLC= pancreas-to-lesion contrast, CNR= contrast-to-noise ratio, N/A= not applicable: pairwise tests were not conducted.

**Table 3:** Visual Score and Lesion Sensitivity with HPAP Images with Partial Downslope Injection Technique and HAP and PPP Images with Standard Injection Technique.

Qualitative Analyses						
				P value		
	HPAP	HAP	PPP	overall	HPAP vs HAP	HPAP vs PPP
Visual score	4 [4], [5]	4 [3], [4], [5]	4 [4], [5]	.30	N/A	N/A
Lesion Sensitivity	43/52 (82.7%)	28/47 (59.6%)	24/31( 77.4%)	.03	.03	.56
Lesion Sensitivity (20 mm or smaller)	10/15( 66.7%)	8/20( 40%)	11/14( 78.6%)	.51	N/A	N/A

Note. --Data presented as median [interquartile range] HPAP= hepatopancreatic arterial-dominant phase, HAP= hepatic arterial-dominant phase, PPP= pancreatic parenchymal phase, N/A= not applicable: pairwise tests were not conducted.

### 3.2 Quantitative evaluation (Table 2)

No statistically significant differences in CT number were observed between the different CT scanners(p = .11). There were no significant differences in the mean CE of the aorta among the three different techniques (p = .92). The mean CE of the pancreatic parenchyma, PLC, and CNR with the HPAP images (95.2 HU, 66.0 HU, and 6.8, respectively) were significantly higher than those with the HAP images (79.8 HU, 53.8 HU, and 5.6, respectively) (p < .01, p = .01, p = .02, respectively) and was similar to those with the PPP images (89.1 HU, 65.1 HU, and 6.5) (p = .09, p = .79, p = .94, respectively). The mean CE of the portal vein with the HPAP images (154.8 HU) was greater than that with both the HAP (132.1 HU) (p < .01) and the PPP images (136.2 HU) (p = .02). There were no significant differences in the mean CE of the lesion among the three different techniques.

### 3.3 Qualitative evaluation (Table 3)

There was no significant difference in the visual scores for the HPAP images (a median of 4 and an interquartile range of 4–5) compared to those for the HAP images (a median of 4 and an interquartile range of 3–5) and PPP images (a median of 4 and an interquartile range of 4–5) (p = .30). However, the lesion sensitivities determined based on the results of the visual score with the HPAP images (43/52, 82.7%) were significantly higher than those with the HAP images (28/47, 59.6%) (p = .03). There were no significant difference in the lesion sensitivity between the HPAP and the PPP images (24/31, 77.4%) (p = .56). In cases with a measurement of 20 mm or smaller, 15 cases of HPAP, 20 cases of HAP and 14 cases of PPP. Of these, 10 cases of HPAP (10/15, 66.7%), 8 cases of HAP (8/20, 40%), and 11 cases of PPP (11/14, 78.6%) were deemed detectable. No statistically significant differences in lesion sensitivity were observed between the three techniques (p = .51), but the sensitivity of HAP tended to be lower than that of HPAP or PPP for lesions measuring 20 mm or smaller.

### 3.4 Interobserver agreement

Fleiss’ kappa values for lesion conspicuity were 0.74 (95% CI: 0.673–0.807), indicating substantial agreement for all image type interpretations.

## 4 Discussion

It has been well known that arterial-phase CE-CT images play a critical role in the detection of pancreatic lesions and hypervascular hepatic lesions. However, with the standard injection technique, the optimal scan timing for detecting these lesions differed: those are the PPP images for the former and the HAP images for the latter. There is considerable controversy surrounding the optimal phase of CE-CT images that radiologists should request when conducting screening examinations for pancreatic or liver lesions. Previous studies have shown that PPP images are effective for detecting PDAC because the contrast enhancement of pancreatic parenchyma reaches its peak during this phase, resulting in greater tumor-to-pancreas contrast and higher CNR [3], [17], [18]. Furthermore, several studies have reported that increased tumor-to-pancreas contrast and CNR improve lesion conspicuity and diagnostic performance for PDAC detection [19], [20]. Consistent with these observations, the present study showed significantly higher pancreatic enhancement, PLC, CNR, and lesion sensitivity on PPP images than on HAP images obtained using the standard injection technique. In the present study, we evaluated the image contrast and the diagnostic performance of the HPAP images which we newly created with the pDiT for detecting PDAC compared to the HAP and the PPP images with the standard injection technique: the present results proved that both the image contrast including the mean CE of the pancreatic parenchyma, PLC and CNR, and the lesion sensitivity with the HPAP images showed significantly higher than those with the HAP images and was similar to those with the PPP images. In cases with a measurement of 20 mm or smaller, no statistically significant differences in lesion sensitivity were observed between the three techniques, but the sensitivity of the HPAP images tended to be higher than that of the HAP images and was comparable to that of the PPP images. Based on the results, the HPAP images with the DiT can solve the above-mentioned dispute because the HPAP images can realize the same diagnostic performance for pancreatic cancers as those of the PPP images despite being obtained at the same scan timing as that of the HAP images with the standard injection technique, resulting in synchronizing arterial-phase imaging protocol of the liver and the pancreas. This finding is clinically important because previous attempts to improve PDAC detectability have mainly focused on optimizing scan timing or adding additional acquisition phases [21], [22], [23]. In contrast, the present study improved contrast enhancement of pancreatic parenchyma and lesion detectability without changing the scan timing, but rather by modifying the contrast injection profile.

For creating such successful HPAP images, two major technical properties of the DiT were essential: using the DiT, the peak CE time and value of the aorta become earlier and greater compared to those with the standard injection technique, thereby those of the pancreatic parenchyma should follow the same pattern as those of the aorta.

In the present study, the diagnostic performance of HPAP images for pancreatic ductal adenocarcinoma, which is a hypovascular lesion, was comparable to that of PPP; therefore, HPAP images did not demonstrate greater utility than PPP images in the evaluation of hypovascular pancreatic tumors. On the other hand, a previous study have shown that the diagnostic performance for hypervascular lesions is improved when using DiT compared to standard injection technique [11]. Therefore, in CT examinations performed for pancreatic tumors of unknown nature, acquiring HPAP images using DiT is considered useful. Since the optimal timing for acquiring HAP images—which are useful for evaluating hypervascular lesions—and PPP images—which are useful for evaluating hypovascular lesions due to maximized contrast enhancement in the pancreatic parenchyma—differs slightly with the standard injection technique, it was necessary to determine which phase to acquire. However, since HPAP imaging allows for obtaining the contrast of both HAP and PPP in a single phase, it has the potential to be a useful imaging method for the screening of pancreatic tumors where hypervascular lesions may be present.

The present study had several limitations. First, the present study was retrospectively investigated in a single institution with a small sample size. Therefore, further multicenter studies with a sufficient sample size should be needed to consolidate the usefulness of the HPAP images with the DiT. Second, hypervascular pancreatic lesions and their hepatic metastases were not evaluated in the present study. Therefore, future studies by including hypervascular pancreatic lesions will be needed although we have already reported that the HAP images with the DiT is useful and shows higher lesion sensitivity in the hypervascular hepatic lesions, in which metastases from pancreatic neuroendocrine neoplasms are included, than those with the standard injection technique. Third, our patients might be smaller (overall mean body weight: 53.4 ± 8.2 kg) than patients in some parts of the world. The application of DiT was constrained by the excessively high initial injection rate, which may represent its principal limitation. In the present study, however, this limitation was not observed in any patient, as the partial DiT had already been refined from the original protocol to mitigate the initial injection rate. Still, excessive increase of the initial injection rate of the partial DiT might possibly be limited in clinical use, especially in heavier patients or those with poor intravenous access. Fourth, all images were acquired at the fixed scan timing after injection of CM without bolus tracking, potentially introducing some variability and bias in the present results. Fifth, the combinations of the iodine dose and the tube voltage were different between the HPAP images with the DiT (360 mgI/kg and 80 kVp) and the HAP and PPP images with the standard injection technique (600 mgI/kg and 120 kVp), although the image contrast should become similar each other between the different two techniques based on the many previous investigations [12], [13]. But this may have influenced the visual assessment of the lesions. Sixth, given that all cases involved PDAC, it was possible to calculate the sensitivity, but not the specificity. In future research, a wider range of cases should be examined in order to assess specificity. Seventh, the choice of injection techniques and imaging phases exhibited variability in the present study, a factor which may have exerted a influence on the results. Eighth, this study lacks within-subject comparisons; future research should employ a design that allows for such comparisons.

In conclusion, we believe that the HPAP images which we newly created with the DiT, are useful as the optimal arterial-phase images in the pancreas because they can provide equivalent image contrast and the lesion sensitivity for the pancreatic cancers as the PPP images with the standard injection technique in spite of being obtained with the same scan timing as that of the HAP images with the standard injection technique, resulting in unifying arterial-phase CE-CT protocols in the liver and the pancreas into one. Since HPAP images allows for obtaining the contrast of both HAP and PPP in a single phase, it has the potential to be a useful imaging modality for the screening of pancreatic tumors.

## CRediT authorship contribution statement

**Hiroyuki Yasui:** Writing – review & editing, Visualization, Data curation. **Tomoaki Ichikawa:** Writing – review & editing, Methodology, Funding acquisition, Conceptualization. **Yoshito Tsushima:** Writing – review & editing, Supervision. **Koji Muroga:** Writing – review & editing, Methodology, Conceptualization. **Takayuki Yokota:** Writing – review & editing, Writing – original draft, Visualization, Formal analysis, Data curation.

## Ethical statement

This study was approved by the Institutional Review Board of Gunma University Hospital. Given the retrospective nature of the study and complete anonymization of the images, the requirement for informed consent was waived, and patients were provided with the opportunity to opt out via the hospital website.

## Funding

This study was supported by Nemoto Kyorindo Co., Ltd. (APC coverage).

## Declaration of Competing interest

The authors declare that they have no known competing financial interests or personal relationships that could have appeared to influence the work reported in this paper.

## Data Availability

Data generated or analyzed during the study are available from the corresponding author by request.

## References

[1] Bilreiro C., Andrade L., Santiago I., Marques R.M., Matos C. (2024). Imaging of pancreatic ductal adenocarcinoma - an update for all stages of patient management. Eur. J. Radiol. Open..

[2] Conroy T., Pfeiffer P., Vilgrain V., Lamarca A., Seufferlein T., O’Reilly E.M., Hackert T., Golan T., Prager G., Haustermans K., Vogel A., Ducreux M. (2023). Pancreatic cancer: ESMO Clinical Practice Guideline for diagnosis, treatment and follow-up. Ann. Oncol..

[3] Singhal S., Prabhu N.K., Sethi P. (2017). S. Moorthy, Role of Multi Detector Computed Tomography (MDCT) in Preoperative Staging of Pancreatic Carcinoma. J. Clin. Diagn. Res. JCDR.

[4] Fletcher J.G., Wiersema M.J., Farrell M.A., Fidler J.L., Burgart L.J., Koyama T., Johnson C.D., Stephens D.H., Ward E.M., Harmsen W.S. (2003). Pancreatic malignancy: value of arterial, pancreatic, and hepatic phase imaging with multi-detector row CT. Radiology.

[5] Tamm E.P., Balachandran A., Bhosale P.R., Katz M.H., Fleming J.B., Lee J.H., Varadhachary G.R. (2012). Imaging of pancreatic adenocarcinoma: update on staging/resectability. Radiol. Clin. North. Am..

[6] Lee K.H.Y., O’Malley M.E., Haider M.A., Hanbidge A. (2004). Triple-Phase MDCT of Hepatocellular Carcinoma. Am. J. Roentgenol..

[7] Kamaya A., Maturen K.E., Tye G.A., Liu Y.I., Parti N.N., Desser T.S. (2009). Hypervascular liver lesions. Semin. Ultrasound CT MR.

[8] Kanematsu M., Goshima S., Kondo H., Nishibori H., Kato H., Yokoyama R., Miyoshi T., Hoshi H., Onozuka M., Moriyama N. (2005). Optimizing scan delays of fixed duration contrast injection in contrast-enhanced biphasic multidetector-row CT for the liver and the detection of hypervascular hepatocellular carcinoma. J. Comput. Assist. Tomogr..

[9] Goshima S., Kanematsu M., Kondo H., Yokoyama R., Miyoshi T., Nishibori H., Kato H., Hoshi H., Onozuka M., Moriyama N. (2006). MDCT of the Liver and Hypervascular Hepatocellular Carcinomas: Optimizing Scan Delays for Bolus-Tracking Techniques of Hepatic Arterial and Portal Venous Phases. Am. J. Roentgenol..

[10] Kondo H., Kanematsu M., Goshima S., Miyoshi T., Shiratori Y., Onozuka M., Moriyama N., Bae K.T. (2007). MDCT of the pancreas: optimizing scanning delay with a bolus-tracking technique for pancreatic, peripancreatic vascular, and hepatic contrast enhancement. AJR Am. J. Roentgenol..

[11] Takayama H., Ichikawa T., Yasui H., Fukushima Y., Fukuda J., Sakai Y., Takeuchi T., Muroga K., Tsushima Y. (2025). Usefulness of linearly-decelerated injection (downslope) method in arterial-phase CT of liver in evaluation of hypervascular lesions compared with standard injection method. Eur. J. Radiol..

[12] Nakaura T., Awai K., Maruyama N., Takata N., Yoshinaka I., Harada K., Uemura S., Yamashita Y. (2011). Abdominal Dynamic CT in Patients with Renal Dysfunction: Contrast Agent Dose Reduction with Low Tube Voltage and High Tube Current–Time Product Settings at 256–Detector Row CT. Radiology.

[13] Nakaura T., Nakamura S., Maruyama N., Funama Y., Awai K., Harada K., Uemura S., Yamashita Y. (2012). Low Contrast Agent and Radiation Dose Protocol for Hepatic Dynamic CT of Thin Adults at 256–Detector Row CT: Effect of Low Tube Voltage and Hybrid Iterative Reconstruction Algorithm on Image Quality. Radiology.

[14] Higaki T., Nakaura T., Kidoh M., Yuki H., Yamashita Y., Nakamura Y., Tatsugami F., Baba Y., Iida M., Awai K. (2018). Effect of contrast material injection duration on arterial enhancement at CT in patients with various cardiac indices: Analysis using computer simulation. PLOS ONE.

[15] Kanda Y. (2013). Investigation of the freely available easy-to-use software “EZR” for medical statistics. Bone Marrow Transplant..

[16] Landis J.R., Koch G.G. (1977). The measurement of observer agreement for categorical data. Biometrics.

[17] Almeida R.R., Lo G.C., Patino M., Bizzo B., Canellas R., Sahani D.V. (2018). Advances in Pancreatic CT Imaging. Am. J. Roentgenol..

[18] Francis I.R. (2003). Role of CT in the detection and staging of pancreatic adenocarcinoma. Cancer Imaging.

[19] He Y.-L., Zhang D.-M., Xue H.-D., Jin Z.-Y. (2013). Clinical Value of Dual-energy CT in Detection of Pancreatic Adenocarcinoma: Investigation of the Best Pancreatic Tumor Contrast to Noise Ratio. Chin. Med. Sci. J. Chung-Kuo Hsueh Ko. Hsueh Tsa Chih.

[20] Noda Y., Takai Y., Asano M., Yamada N., Seko T., Kawai N., Kaga T., Miyoshi T., Hyodo F., Kato H., Matsuo M. (2023). Comparison of image quality and pancreatic ductal adenocarcinoma conspicuity between the low-kVp and dual-energy CT reconstructed with deep-learning image reconstruction algorithm. Eur. J. Radiol..

[21] Fukukura Y., Kumagae Y., Fujisaki Y., Yamagishi R., Nakamura S., Kamizono J., Nakajo M., Kamimura K., Nagano H., Takumi K., Yoshiura T. (2021). Adding Delayed Phase Images to Dual-Phase Contrast-Enhanced CT Increases Sensitivity for Small Pancreatic Ductal Adenocarcinoma. AJR Am. J. Roentgenol..

[22] Tang A., Billiard J.-S., Chagnon D.-O., Rizk F., Olivié D., Turcotte S., Chagnon M., Lepanto L. (2014). Optimal Pancreatic Phase Delay with 64-Detector CT Scanner and Bolus-tracking Technique. Acad. Radiol..

[23] Goshima S., Kanematsu M., Kondo H., Yokoyama R., Miyoshi T., Kato H., Tsuge Y., Shiratori Y., Hoshi H., Onozuka M., Moriyama N., Bae K.T. (2006). Pancreas: Optimal Scan Delay for Contrast-enhanced Multi–Detector Row CT. Radiology.

